# SMC4, a novel tumor prognostic marker and potential tumor therapeutic target

**DOI:** 10.3389/fonc.2023.1117642

**Published:** 2023-03-17

**Authors:** Zonglei Zhao, Xixiu Wang, Yan Ding, Xuefeng Cao, Xingyuan Zhang

**Affiliations:** ^1^ Department of Hepatobiliary Surgery, Binzhou Medical University Hospital, Binzhou, Shandong, China; ^2^ Department of Cardiovascular Diseases, Binzhou Medical University Hospital, Binzhou, Shandong, China

**Keywords:** SMC4, tumor, prognosis, biomarker, targeted therapy, overall survival

## Abstract

The structural maintenance of chromosome 4 (SMC4) is a member of the ATPase family of chromosomes. The most widely reported function of SMC4, as well as the remaining subunits of whole condensin complexes, is compression and dissociation of sister chromatids, DNA damage repair, DNA recombination, and pervasive transcription of the genome. Studies have also shown that SMC4 plays an exceedingly essential role in the division cycle of embryonic cells, such as RNA splicing, DNA metabolic process, cell adhesion, and extracellular matrix. On the other hand, SMC4 is also a positive regulator of the inflammatory innate immune response, while excessive innate immune responses not only disrupt immune homeostasis and may lead to autoimmune diseases, but even cancer. To further understand the expression and prognostic value of SMC4 in tumors, we provide an in-depth review of the literature and several bioinformatic databases, for example, The Cancer Genome Atlas (TCGA), Genotype-Tissue Expression (GTEx), Clinical Proteomic Tumor Analysis Consortium (CPTAC), The Human Protein Atlas and Kaplan Meier plotter tools, illustrating that SMC4 plays a vital role in the occurrence and development of tumors, and high expression of SMC4 seems to consistently predict worse overall survival. In conclusion, we present this review which introduces the structure, biological function of SMC4, and its correlation with the tumor in detail; it might provide new insight into a novel tumor prognostic marker and potential tumor therapeutic target.

## 1 Introduction

Structural maintenance of chromosome 4 (SMC4), a member of the chromosome ATPase family, is a class of proteins evolutionarily conserved from bacteria to humans (1). It forms a chromosome pentamer complex with homologous protein structural maintenance of chromosome 2 (SMC2) and other non-structural maintenance of chromosome (SMC) subunits, called condensin. The pentamer complex plays an essential role in regulating chromatin dynamics, sister chromatid condensation, chromatin condensation, DNA replication, DNA repair, and transcription (2). Furthermore, SMC4 is also a vital eukaryotic SMC protein related to cell survival and death. Numerous pieces of evidence suggest that SMC4 can promote cell proliferation in various tumors by regulating the division cycle of embryonic cells (3). In addition, recent research has also revealed that SMC4 participates in inflammatory innate immune responses through enhancing TLR and virus-triggered activation of NF-κB and IRF3, leading to the production of proinflammatory cytokines and IFN-β. Excessive innate immune responses not only disrupt immune homeostasis and may lead to autoimmune diseases but even cancer, where the development mechanism may be strongly related to the alteration of the tumor microenvironment (TME) (4), which consists mainly of fibroblasts, adipocytes, adaptive, endothelial cells, neurons, and innate immune cells, as well as its non-cellular components, such as cytokines, growth factors, chemokines, and extracellular vesicles. Furthermore, our bioinformatics analysis has also shown that SMC4 has changed gene and protein expression levels in various tumors and plays a critical regulatory role in the occurrence, development, and prognosis of tumors. This paper review introduces the structure and biological function of SMC4 and its correlation with tumors. It is surprising to find that SMC4 may be a novel tumor prognostic marker and potential tumor therapeutic target.

## 2 The structure of SMC4

**Figure 1:**
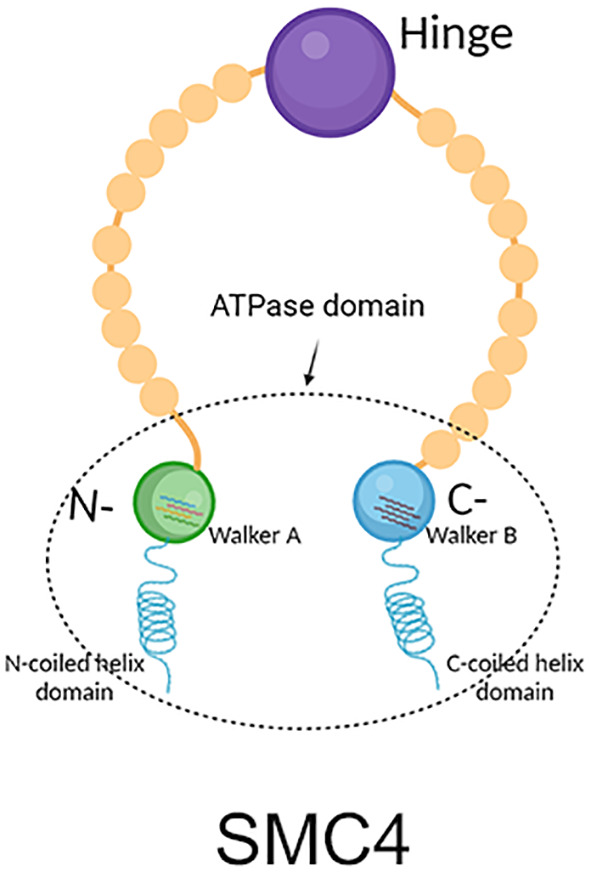
Structure diagram of SMC4, which contains mainly five different domains. The green circle represents the N-terminal, which contains Walker A the blue circle represents the C-terminal, which contains Walker B the purple circle represents the hinge; the helix on the left represents the N-terminal coiled helix domain; the helix on the right represents the C-terminal coiled helix domain. The C-terminal and N-terminal are close to each other to form the ATPase domain.

As a member of the SMC family, SMC4 protein is evolutionarily conserved. SMC proteins from bacteria to humans have basic structures and contain five different domains, and SMC4 is no exception. SMC4 protein is basically made up of 1000 to 1400 amino acids, with a molecular weight of 110-170 kDa. It is made up of a moderately conserved hinge region, highly conserved N-and C-terminal domains, and a coiled helix domain with N-terminal and C-terminal connections respectively (5, 6). Among them, the C-terminal domain contains about 150 amino acids, including Walker B motif, and the N-terminal domain contains about 160 amino acids, with Walker a motif. Both Walker A and Walker B motifs are a conserved sequence of NTP binding amino acids and were discovered and described first by J.E. Walker (7). In addition, SMC4 molecules usually reverse fold in the hinge domain, making its two double helix domains parallel to each other. Then the C-terminal and N-terminal are close to each other to form a rod-shaped ATPase domain, which is usually similar to the ATP binding cassette (ABC) domain (**Figure 1**) (8).

## 3 Biological function of SMC4

**Figure 2:**
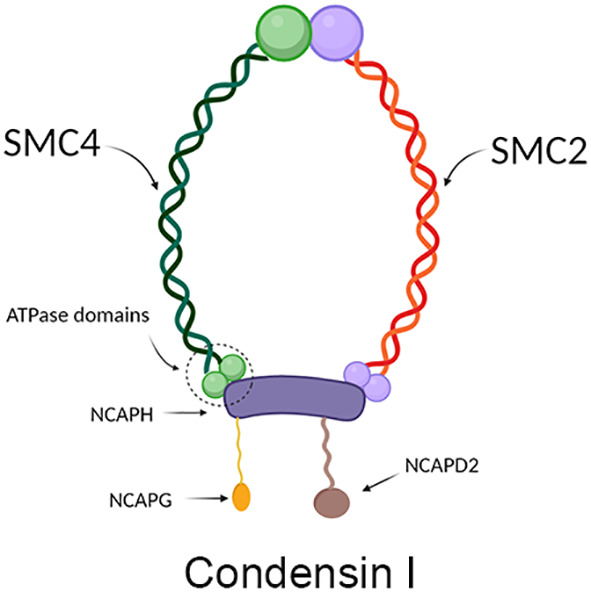
Structure diagram of condensin I, which contains mainly condensin, NCAPH, NCAPD2, and NCAPG. The green part on the left represents SCM4, and the red part on the right represents SMC2, which together form condensin; the purple protein in the middle represents NCAPH, the yellow protein in the lower left represents NCAPG, and the brown protein in the lower right represents NCAPD2.

**Figure 3:**
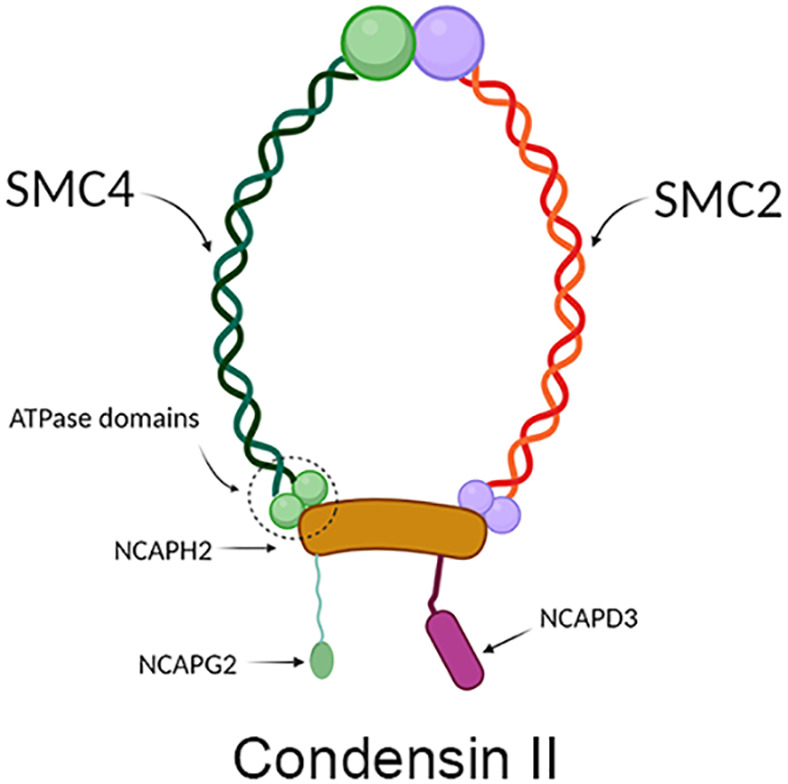
Structure diagram of condensin II, which contains mainly condensin, NCAPH2, NCAPD3, and NCAPG2. The green part on the left represents SCM4, and the red part on the right represents SMC2, which together form condensin; the yellow protein in the middle represents NCAPH2, the green protein on the lower left represents NCAPG2, and the pink protein on the lower right represents NCAPD3.

SMC4 protein is one of the members of the ATPase superfamily. It is involved in forming the core subunit of the condensin complex by binding to the SMC2 monomer in the hinge region to form a V-shaped structure (6). In addition to the SMC2 and SMC4 core subunits, condensin I complexes also contain three non-SMC subunits: non-SMC condensin I complex subunit H (NCAPH), non-SMC condensin I complex subunit D2 (NCAPD2) and non-SMC condensin I complex subunit G (NCAPG) (**Figure 2**), while in condensin II these are NCAPH2, NCAPG2 and NCAPD3 (**Figure 3**) (9). The condensin complex can promote the correct compression and dissociation of sister chromatids through its intramolecular bridging effect in an ATP hydrolysis-dependent manner. Meanwhile, the SMC4 protein plays an essential role in regulating the ATPase domain (8). In contrast to SMC2 protein, SMC4 protein has a high affinity for APT enzymes; therefore, it can be regarded as a “switch” of the condensin complex (9). In addition, a growing body of research showed that SMC4 plays an exceedingly essential role in the division cycle of embryonic cells, for example, RNA splicing, DNA metabolic process, cell adhesion, and extracellular matrix (10). Therefore, SMC4 protein can play a vital role in promoting cell proliferation (11). Furthermore, Wang Q et al. found that SMC4 promotes an inflammatory innate immune response, which promotes innate activation of NF-κB and IRF3 by enhancing the transcription of NEMO, and subsequently inducing the activation of proinflammatory cytokines and type I interferon genes (12).

## 4 Correlation between SMC4 and various tumors (How to infer that it may be a novel tumor prognostic marker and potential tumor therapeutic target)

**Figure 4:**
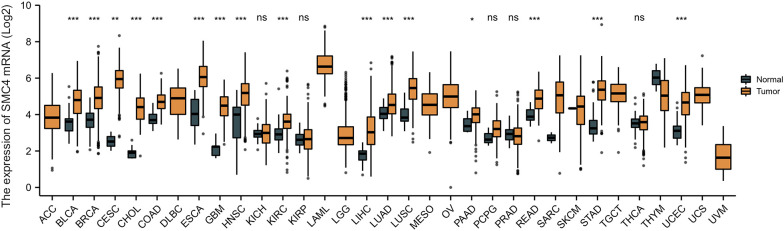
Expression levels of SMC4 mRNA in different types of tumors in TCGA database. Adrenocortical carcinoma (ACC), bladder urothelial carcinoma (BLCA), breast invasive carcinoma (BRCA), cervical squamous cell carcinoma and endocervical adenocarcinoma (CESC), cholangiocarcinoma (CHOL), colon adenocarcinoma (COAD), lymphoid neoplasm diffuse large B-cell lymphoma (DLBC), esophageal carcinoma (ESCA), glioblastoma multiforme (GBM), head and neck squamous cell carcinoma (HNSC), kidney chromophobe (KICH), kidney renal clear cell carcinoma (KIRC), kidney renal papillary cell carcinoma (KIRP), acute myeloid leukemia (LAML), brain lower grade glioma (LGG), liver hepatocellular carcinoma (LIHC), lung adenocarcinoma (LUAD), lung squamous cell carcinoma (LUSC), mesothelioma (MESO), ovarian serous cystadenocarcinoma (OV), pancreatic adenocarcinoma (PAAD), pheochromocytoma and paraganglioma (PCPG), prostate adenocarcinoma (PRAD), rectum adenocarcinoma (READ), sarcoma (SARC), skin cutaneous melanoma (SKCM), stomach adenocarcinoma (STAD), testicular germ cell tumors (TGCT), thyroid carcinoma (THCA), thymoma (THYM), uterine corpus endometrial carcinoma (UCEC), uterine carcinosarcoma (UCS), uveal melanoma (UVM); TCGA, The Cancer Genome Atlas. ns, *p* > 0.05; *p < 0.05; **p < 0.01; ***p < 0.001.

As we all know, tumors, especially malignant tumors, have biological characteristics, including proliferation and abnormal cell differentiation, uncontrolled growth, decreased apoptosis, easy invasion, and metastasis. Their occurrence and development is an enormously complex process of multi factors and multi-steps. Genomic instability refers to the increased tendency to accrue genomic alterations, which drives heterogeneity and is a hallmark of cancer. At the same time, an aberration in the DNA repair mechanisms may result in genomic and chromosomal instability and the accumulation of mutations. The activation of oncogenes or the inactivation of tumor suppressor genes is a dire consequence of genomic instability that even may bring the cells into a cancerous phenotype (13). SMC4 protein, as a sensitive switch of the condensin complex, which combines with SMC2 monomer to form a V-shaped structure, and then combines with other non-SMC subunits to form the condensin complex, plays a highly crucial role in the process, including DNA repair and correct compression and dissociation of sister chromatids; therefore SMC4 is inextricably related to the occurrence and development of tumor. Moreover, a study reported that tumors could be conceived of particular “organs” because various signaling, transcriptional and metabolic pathways are shared between organogenesis and malignant tumors (10). A growing number of studies have shown that SMC4 also plays an exceedingly crucial role in the division cycle of embryonic cells, such as RNA splicing, DNA metabolic processes, cell adhesion, and extracellular matrix. Likewise, tumor cells with high SMC4 expression may mimic the gene expression patterns of embryonic cells to enhance their competitive advantage over normal somatic cells (11, 14). On the other hand, recent research has also revealed that SMC4 participates in inflammatory innate immune responses through enhancing TLR and virus-triggered activation of NF-κB and IRF3, leading to the production of proinflammatory cytokines and IFN-β (12). An excessive innate immune response not only disrupts the immune balance, leading to autoimmune diseases but also even results in cancer. The development mechanism may be strongly related to the fact that immune cell infiltration and secretion of cytokine promote the formation of the TME, thus facilitating tumor progression, metastasis, and therapeutic resistance (1). In order to further a more comprehensive understanding of SMC4 and the potential relationship between SMC4 and tumors, from The Cancer Genome Atlas (TCGA) database, the pan-cancer analysis showed that the expression of SMC4 mRNA was highly expressed in most types of cancers, such as breast invasive carcinoma (BRCA), colon adenocarcinoma (COAD), cervical squamous cell carcinoma and endocervical adenocarcinoma (CESC), glioblastoma multiforme (GBM), liver hepatocellular carcinoma (LIHC), pancreatic adenocarcinoma (PAAD), lung adenocarcinoma (LUAD), lung squamous cell carcinoma (LUSC) and rectum adenocarcinoma (READ) (**Figure 4**). A large number of studies also demonstrate that patients with high SMC4 expression tend to have a poor prognosis (14–47). In summary, all of these indicate that SMC4 may be enormously associated with the occurrence, development, evolution, and prognosis of tumors and even become a new tumor prognostic marker and potential therapeutic target (10, 13).

### 4.1 SMC4 and primary liver cancer

**Figure 5:**
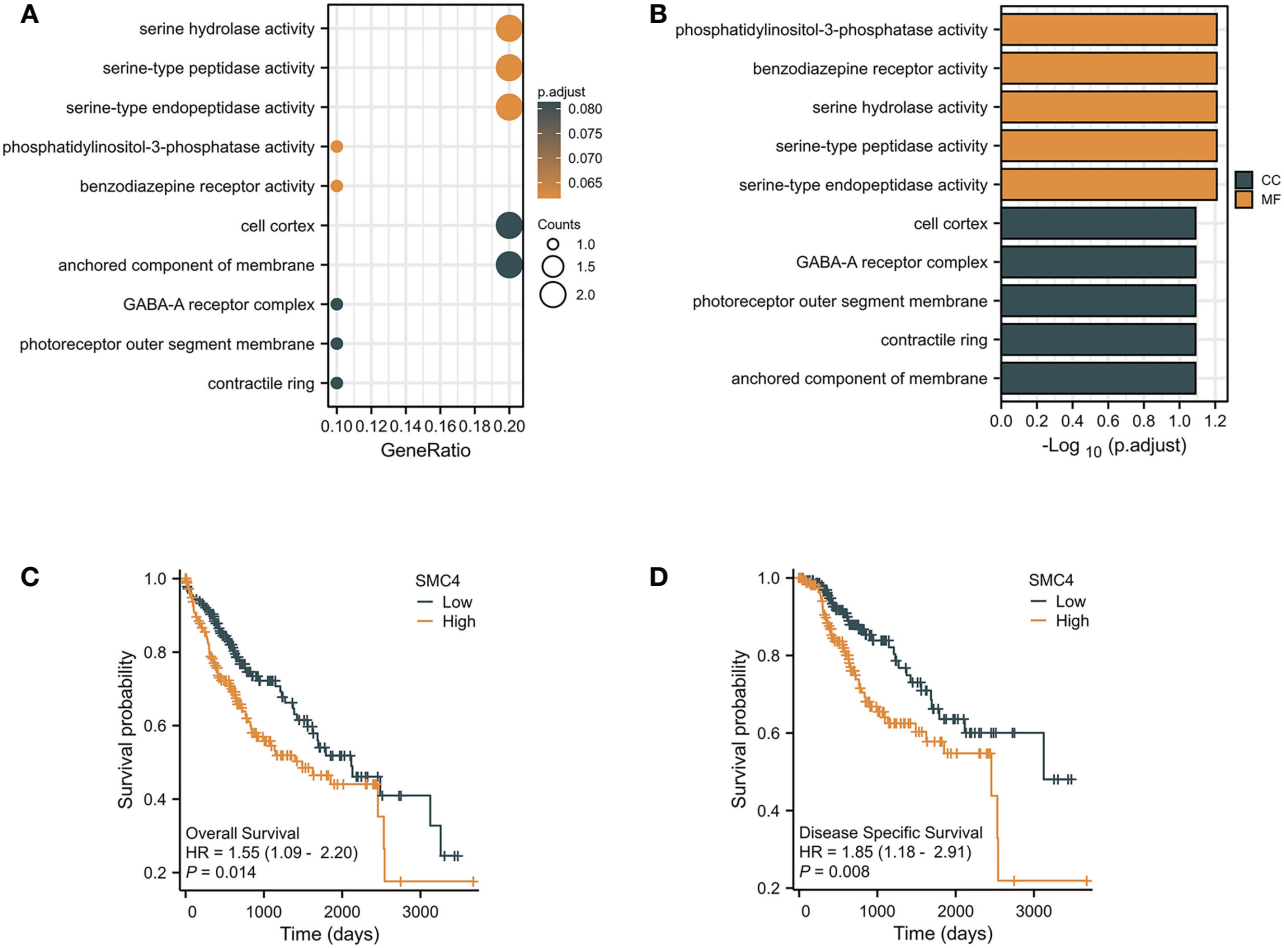
The enrichment analysis and prognostic value of SMC4 in HCC. **(A, B)** Biological process enrichment related to SMC4-related genes in the TCGA database. **(C, D)** The prognostic value of SMC4 in OS and DSS of HCC in the TCGA database. TCGA, The Cancer Genome Atlas; OS, overall survival; DSS, disease-specific survival (DSS). CC, cellular component; MF, molecular function; HR, Hazard Ratio.

The occurrence and development of primary liver cancer is an exceedingly complex, long-term, multi-factor, multi-step gradual evolution process. Searching for new tumor markers and exploring the intervention targets of tumor-targeted therapy has become a hot research topic at home and abroad in recent years (15). *In vitro*, the results of the cell invasion experiment, scratch repair experiment, and cell clone formation experiment of liver cancer cells confirmed that the migration rate of liver cancer cells transfected with SMC4 interference vector group decreased significantly, and the proliferation rate decreased from 100% to 73.4%, suggesting that SMC4 plays an exceedingly critical role in promoting the proliferation and invasion of liver cancer cells (16). Its mechanism may be related to miR-219-SMC4-JAK2/STAT3 (tyr705), a new regulatory pathway in primary liver cancer (17). Moreover, research also found that under hypoxic culture, through HIF-1-miR-219-SMC4 regulatory pathway suppression of SMC4 could inhibit the proliferation rate of hepatocellular carcinoma (HCC) cells through inducing G1 phase arrest and affecting cell migration ability by affecting the process of epithelial-to-mesenchymal transition (EMT) (18). Through real-time polymerase chain reaction (PCR), Western blot, immunohistochemistry (IHC), and other experimental results, SMC4 expression has been confirmed up-regulated in HCC tissues, and the expression level of SMC4 in HCC tissues was associated with tumor size, TNM stage, cell differentiation, and vascular invasion, indicating that the expression level of SMC4 is related to the poor prognosis of patients with hepatoma (16). Furthermore, GO analysis indicated that SMC4 had significant regulation on serine hydrolase activity, serine-type peptidase activity, serine-type endopeptidase activity, phosphatidylinositol-3-phosphatase activity, anchored component of membrane, contractile ring, photoreceptor outer segment membrane, GABA-A receptor complex, cell cortex and benzodiazepine receptor activity (**Figures 5A, B**). The correlation between SMC4 expression and the prognosis of patients with HCC was calculated using the Kaplan Meier method. HCC patients were divided into high and low expression groups based on the SMC4 expression median value. Compared with the low SMC4 expression group, both the overall survival (OS) and disease-specific survival (DSS) of the high SMC4 expression group exhibited a significantly worse prognosis (HR = 1.55 (1.09–2.20), *p* = 0.014) and DSS (HR = 1.85 (1.18–2.91), *p* = 0.008) (**Figures 5C, D**). These results indicate that SMC4 genes could significantly enrich our understanding of the development and recurrence of HCC and could be therapeutic targets for HCC treatment (19).

### 4.2 SMC4 and lung cancer

**Figure 6:**
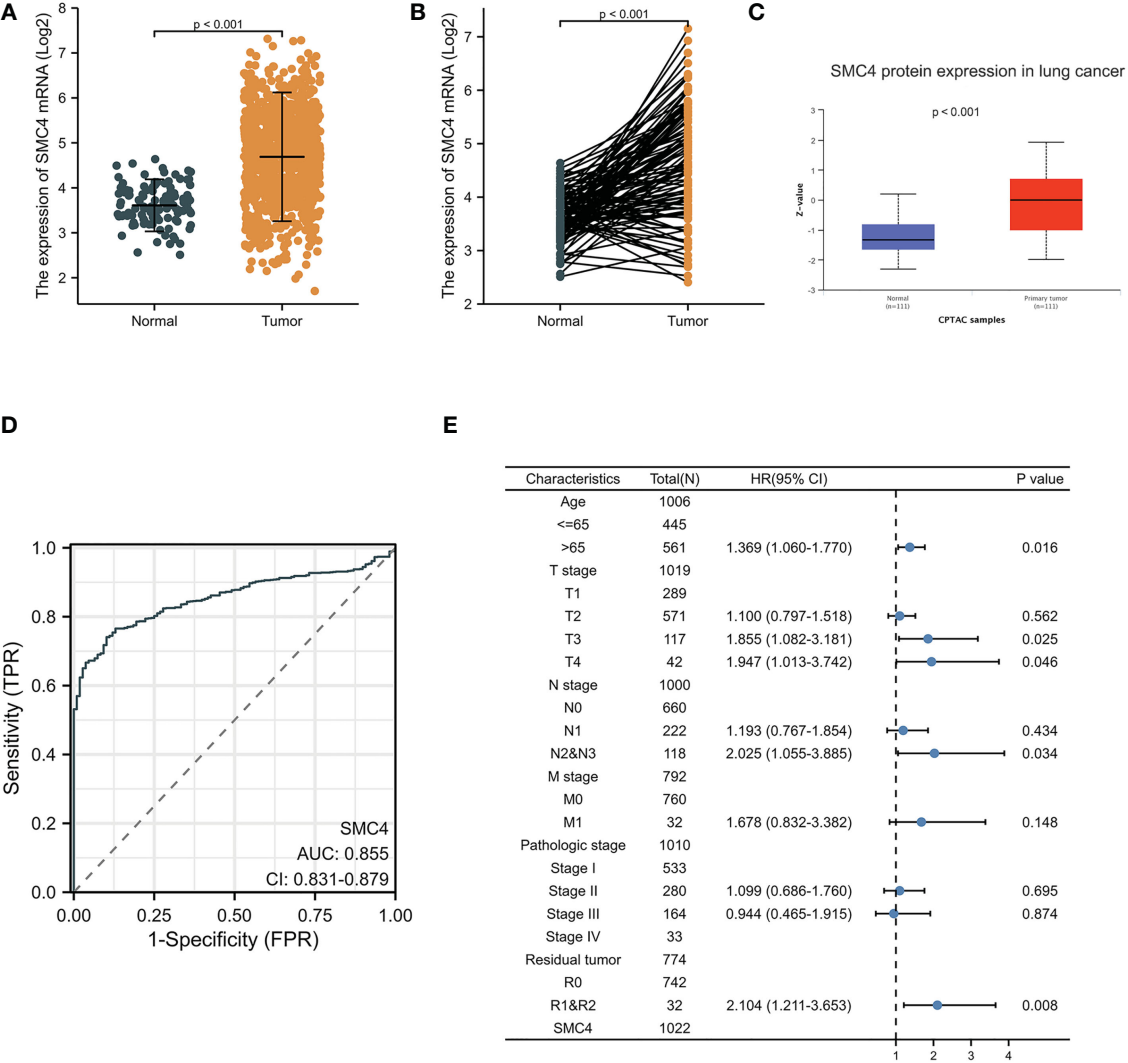
Expression levels of SMC4 mRNA and SMC4 protein and the prognostic values of SMC4 expression in patients with lung cancer. **(A)** Expression of SMC4 mRNA in lung cancer and non-matched normal tissues in the TCGA databases. **(B)** Expression of SMC4 mRNA in lung cancer and matched normal tissues in the TCGA databases. **(C)** Expression of SMC4 protein in lung cancer in the CPTAC databases. **(D)** ROC curves for classifying lung cancer versus normal lung tissues in the TCGA database. **(E)** Forest map based on multivariate Cox analysis for overall survival in the TCGA databases. TCGA, The Cancer Genome Atlas; CPTAC, Clinical Proteomic Tumor Analysis Consortium; ROC, receiver operating characteristic.

Lung cancer, which is known as the most lethal cancer, has the highest incidence and mortality among all kinds of tumors, and its incidence rate is still rising. A 2013 study on genome-scale co-expression network found that SMC4 gene plays an enormously crucial role in the progression cell cycle and is involved in the occurrence and development of lung adenocarcinoma (20). Subsequently, Zhang C et al. concluded that SMC4 plays a crucial role in lung development and tumor formation through co-expression network analysis and gene cluster penetration clustering, which is mainly reflected in cell cycle, cell adhesion, and RNA processing (14). Bioinformatics analysis manifested that the unpaired (*p* < 0.001) and paired (*p* < 0.001) differential expression analyses between normal and lung cancer groups indicated that SMC4 was expressed significantly higher in tumors compared to normal tissue (**Figures 6A, B**). From the Clinical Proteomic Tumor Analysis Consortium (CPTAC) database, we observed that the lung cancer tumor tissues exhibited a significantly higher level of SMC4 protein than that in the normal tissues (*p* < 0.001) (**Figure 6C**). The receiver operating characteristic (ROC) curve indicated that SMC4 expression had good predictive power with an area under the curve (AUC) of 0.855 (95% confidence interval [CI] = 0.831–0.879) to discriminate breast cancer tissues from normal tissues (**Figure 6D**). In the Cox regression model, univariate Cox regression indicates that the T stage (*p* < 0.05), N stage (*p* < 0.001), M stage (*p* < 0.001), SMC4 (*p* = 0.049), pathologic stage (*p* < 0.001), residual tumor (*p* < 0.001) and Age (*p* = 0.022) were correlated with the lousy prognosis of lung cancer (**Table 1**). All variables in univariate Cox regression were included in multivariate Cox regression. Multivariate Cox regression showed that T stage (*p* < 0.05), N stage (*p* < 0.05), Age (*p* = 0.016), and residual tumor (*p* = 0.008) were independent prognostic factors for OS (**Figure 6E**). As a result, we can easily conclude from the above bioinformatic analysis that SMC4 is highly expressed in lung cancer and is tightly linked to lung cancer prognosis. Furthermore, SMC4 gene knockout can significantly inhibit the proliferation and invasion of A549 cells (14); therefore, SMC4 may also be a potential biomarker of lung cancer and help to clarify its carcinogenic mechanism. In the study on the relationship between SMC4 and non-small cell lung cancer, Nie H also confirmed that SMC4 was highly expressed in lung cancer cells and tissues by quantitative reverse-transcription polymerase chain reaction (QRT-PCR), WB, and plasmid transfection. After the overexpression of SMC4 protein, the proliferation ability of PC9 cells and A549 cells were significantly increased, and the expression of cyclin, which is a particularly important positive regulation of the cell cycle considered to be closely related to the occurrence, development, and prognosis of the tumor was significantly up-regulated in cells (21, 22).

**Table 1:** Univariate and multivariate analyses of clinical pathological parameters in patients with lung cancer from TCGA database.

Characteristics	Total (N)	Univariate analysis		Multivariate analysis	
		Hazard ratio (95% CI)	*P* value	Hazard ratio (95% CI)	*P* value
Age	1006				
<=65	445	Reference			
>65	561	1.265 (1.034-1.548)	**0.022**	1.369 (1.060-1.770)	**0.016**
T stage	1019				
T1	289	Reference			
T2	571	1.404 (1.096-1.799)	**0.007**	1.100 (0.797-1.518)	0.562
T3	117	2.258 (1.614-3.161)	**<0.001**	1.855 (1.082-3.181)	**0.025**
T4	42	2.750 (1.760-4.296)	**<0.001**	1.947 (1.013-3.742)	**0.046**
N stage	1000				
N0	660	Reference			
N1	222	1.539 (1.224-1.937)	**<0.001**	1.193 (0.767-1.854)	0.434
N2&N3	118	2.036 (1.537-2.697)	**<0.001**	2.025 (1.055-3.885)	**0.034**
M stage	792				
M0	760	Reference			
M1	32	2.269 (1.439-3.577)	**<0.001**	1.678 (0.832-3.382)	0.148
Pathologic stage	1010				
Stage I	533	Reference			
Stage II	280	1.611 (1.271-2.042)	**<0.001**	1.099 (0.686-1.760)	0.695
Stage III	164	2.262 (1.750-2.924)	**<0.001**	0.944 (0.465-1.915)	0.874
Stage IV	33	3.108 (1.969-4.906)	**<0.001**		
Residual tumor	774				
R0	742	Reference			
R1&R2	32	2.953 (1.847-4.721)	**<0.001**	2.104 (1.211-3.653)	**0.008**
SMC4	1022	1.040 (0.945-1.144)	**0.049**		

TCGA, The Cancer Genome Atlas.

Bold values means statistically significant (p < 0.05).

### 4.3 SMC4 and breast cancer

**Figure 7:**
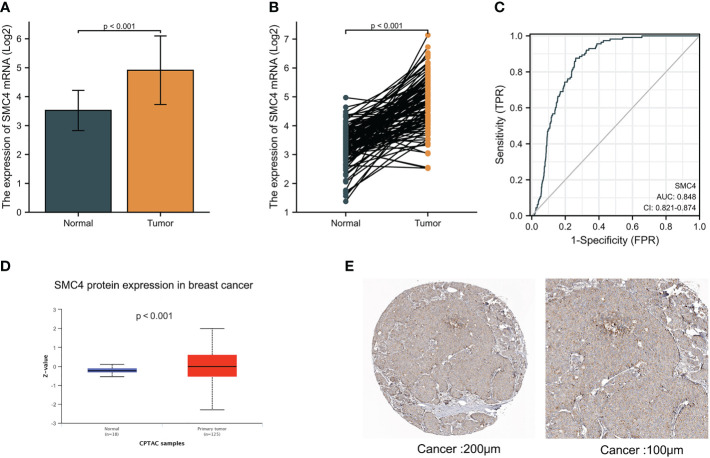
Expression levels of SMC4 mRNA and SMC4 protein in breast cancer. **(A)** Expression of SMC4 mRNA in breast cancer and non-matched normal tissues in the TCGA and GTEx databases. **(B)** Expression of SMC4 mRNA in breast cancer and matched normal tissues in the TCGA and GTEx databases. **(C)** ROC curves for classifying breast cancer versus normal breast tissues in the TCGA database. **(D)** Expression of SMC4 protein level in breast cancer in the CPTAC database. **(E)** SMC4 protein level in breast cancer using immunohistochemistry was analyzed using The Human Protein Atlas database. TCGA, The Cancer Genome Atlas; GTEx, Genotype Tissue Expression Project; CPTAC, Clinical Proteomic Tumor Analysis Consortium; ROC, receiver operating characteristic.

**Figure 8:**
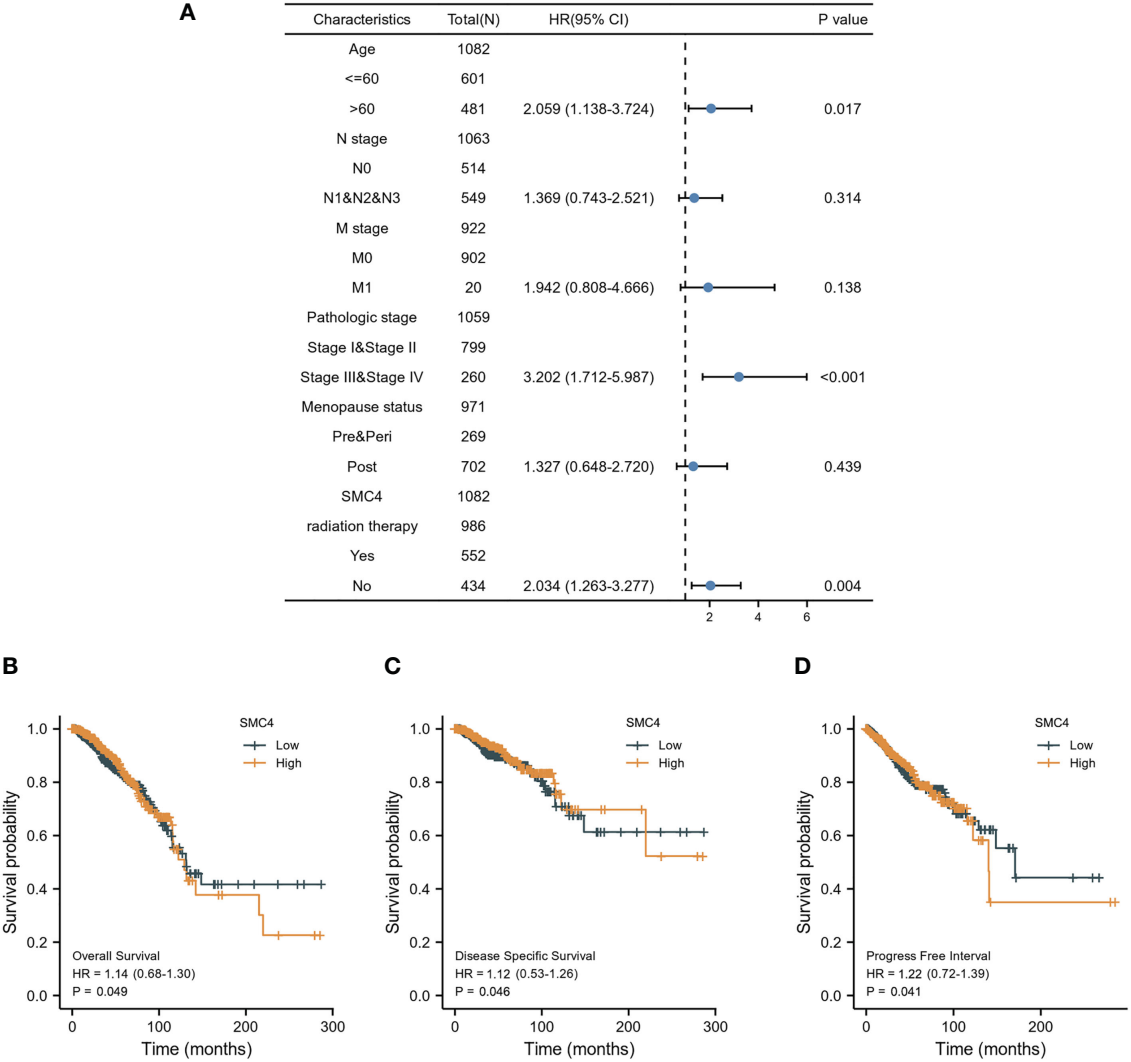
Prognostic values of SMC4 expression in patients with breast cancer. **(A)** Forest map based on multivariate Cox analysis for overall survival in the TCGA databases. Overall survival **(B)**, disease-specific survival **(C)**, and progress-free interval **(D)** for breast cancer patients with high versus low SMC4 in the TCGA databases. TCGA, The Cancer Genome Atlas; OS, overall survival; DSS, disease specific-survival (DSS); progress-free interval, PFI; CI, confidence interval.

Breast cancer (BC), as one of the most common malignant tumors, is a major cause of death in women worldwide. Exploring the potential mechanism of tumor occurrence and development and finding new therapeutic targets has been a hot topic in the diagnosis and treatment of BC. As early as 2005, Wang Y et al. published their research results in Lancet, confirming that SMC4 gene plays an essential role in the cell cycle, DNA replication, recombination and repair, cell assembly, and other vital nodes of BC (23). Kulawiec M et al. later proposed in 2008 that the expression change of SMC4 can affect the chromosome stability of human breast epithelial cells through the p53 network and then produce tumorigenic transformation (24). By introducing the evaluation model of BC susceptibility genes based on multi-objective evaluation, the weighted sum method and the approximate ideal sorting method were also used to calculate and confirm that SMC4 is a potential BC susceptibility gene (25). Bioinformatics analysis demonstrated that the unpaired (*p* < 0.001) and paired (*p* < 0.001) differential expression analyses between normal and breast cancer groups indicated that SMC4 was expressed significantly higher in tumors compared to normal tissue (**Figures 7A, B**). ROC showed that the expression of SMC4 mRNA in BC was 0.848 (95% CI: 0.821–0.874) (**Figure 7C**). Based on the CPTAC database, SMC4 protein level was elevated in breast cancer compared with normal samples (*p* < 0.001) (**Figure 7D**). The immunohistochemistry results from The Human Protein Atlas database showed that SMC4 protein level was clearly enhanced in BC tissues (**Figure 7E**). In the Cox regression model, univariate Cox regression indicates that the Age (*p* < 0.001), N stage (*p* < 0.001), M stage (*p* < 0.001), SMC4 (*p* = 0.036), pathologic stage (*p* < 0.001), menopause status (*p* < 0.001) and radiation therapy (*p* = 0.004) were correlated with the lousy prognosis of lung cancer (**Table 2**). All variables in univariate Cox regression were included in multivariate Cox regression. Multivariate Cox regression showed that Age (*p* = 0.017), pathologic stage (*p* < 0.001), and radiation therapy (*p* = 0.004) were independent prognostic factors for OS (**Figure 8A**). The correlation between SMC4 expression and the prognosis of patients with BC was calculated using the Kaplan Meier method. BC patients were divided into high and low expression groups based on the SMC4 expression median value. The high expression group has a strong correlation with worse OS (HR = 1.14 (0.68–1.30), *p* = 0.049), DSS (HR = 1.12 (0.53–1.26), *p* = 0.046), and progress-free interval (PFI) (HR = 1.22 (0.72–1.39), *p* = 0.041) (**Figures 8B–D**). Moreover, subsequent studies confirmed that interference with SMC4 mRNA expression effectively inhibited the proliferation, migration, and invasive ability of breast cancer MDA-MB-231 cells, with the mechanism possibly related to the activation of the PI3K/AKT signaling pathway (26–28). These studies and bioinformatics analysis results indicate that SMC4 mRNA level is an excellent prognostic biomarker for BC patients, and SMC4 will be a novel tumor prognostic marker and potential tumor therapeutic target.

**Table 2:** Univariate and multivariate analyses of clinical pathological parameters in patients with breast cancer from TCGA database.

Characteristics	Total (N)	Univariate analysis		Multivariate analysis	
		Hazard ratio (95% CI)	*P* value	Hazard ratio (95% CI)	*P* value
Age	1082				
<=60	601	Reference			
>60	481	2.020 (1.465-2.784)	**<0.001**	2.059 (1.138-3.724)	**0.017**
N stage	1063				
N0	514	Reference			
N1&N2&N3	549	2.239 (1.567-3.199)	**<0.001**	1.369 (0.743-2.521)	0.314
M stage	922				
M0	902	Reference			
M1	20	4.254 (2.468-7.334)	**<0.001**	1.942 (0.808-4.666)	0.138
Pathologic stage	1059				
Stage I&Stage II	799	Reference			
Stage III&Stage IV	260	2.391 (1.703-3.355)	**<0.001**	3.202 (1.712-5.987)	**<0.001**
Menopause status	971				
Pre&Peri	269	Reference			
Post	702	2.348 (1.428-3.860)	**<0.001**	1.327 (0.648-2.720)	0.439
SMC4	1082	1.081 (0.931-1.256)	**0.036**		
Radiation therapy	986				
Yes	552	Reference			
No	434	1.737 (1.189-2.536)	**0.004**	2.034 (1.263-3.277)	**0.004**

Bold values means statistically significant (p < 0.05).

### 4.4 SMC4 and colorectal cancer

**Figure 9:**
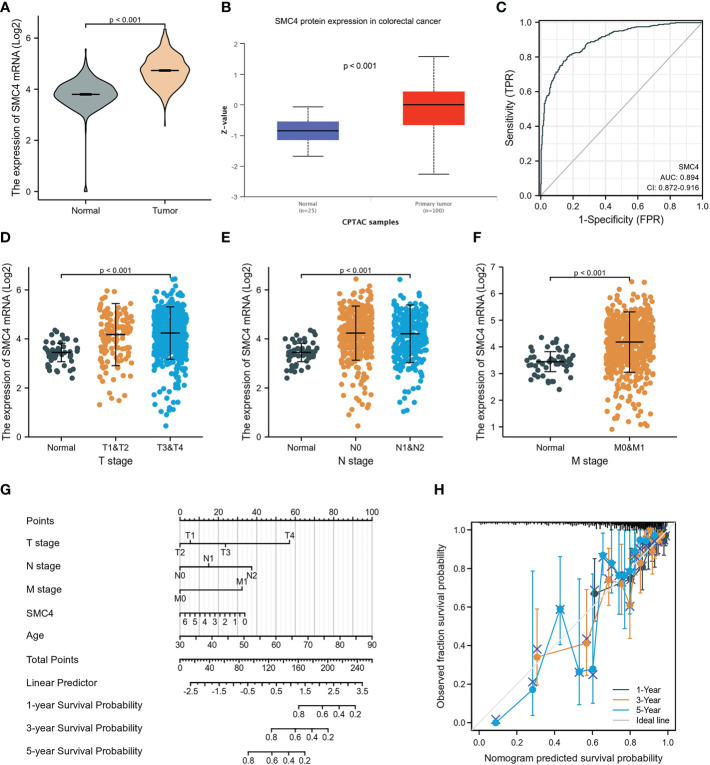
Expression levels of SMC4 and the prognostic values of SMC4 expression in CRC patients, as well as the associations between SMC4 expression and clinicopathological characteristics. **(A)** Expression of SMC4 mRNA in CRC and non-matched normal tissues in the TCGA databases. **(B)** Expression of SMC4 protein in CRC in the CPTAC databases. **(C)** ROC curves for classifying colorectal cancer versus normal colorectal tissues in the TCGA database. Association between the SMC4 expression and the T stage **(D)**, N stage **(E)**, and M stage **(F)**. **(G)** A nomogram that integrates SMC4 and other prognostic factors in SMC4 from TCGA data. **(H)** The calibration curve of the nomogram. TCGA, The Cancer Genome Atlas; CPTAC, Clinical Proteomic Tumor Analysis Consortium.

Colorectal cancer (CRC) is another most common malignant tumor in human beings. Finding the key gene target for the occurrence, development, and prognosis of CRC is an extremely urgent event. Research has found that constantly evolving chromosome complements, or chromosome instability (CIN), is a form of genome instability implicated in the development and progression of CRC. Following SMC4 gene silencing, quantitative imaging microscopy identified increases in CIN-associated phenotypes, including micronucleus formation, changes in nuclear areas, and chromosome numbers, resulting in a significant driver effect of CRC (29). The driving effect can be revealed by LINC-ROR/miR-6833-3p/SMC4 regulatory network (30). In addition, bioinformatics analysis based on TCGA and CPTAC databases indicated that the expression of SMC4 mRNA and protein was significantly higher in CRC tissues than in normal tissues (*p* < 0.001) (**Figures 9A, B**). The ROC curve indicated that the expression of SMC4 mRNA in CRC was 0.894 (95% CI: 0.872–0.916), with a good predictive power to discriminate CRC tissues from normal tissues (**Figure 9C**). Kruskal-Wallis test, followed by the Bonferroni correction, proved that high expression of SMC4 was significantly associated with T stage (*p* < 0.001), N stage (*p* < 0.001), and M stage (*p* < 0.001) (**Figures 9D–F**). To predict the prognosis of patients with CRC, we constructed a nomogram of OS to integrate SMC4 and other prognostic factors, including T classification, N classification, M classification, and age (**Figure 9G**). On the nomogram, a higher total number of points was associated with a worse prognosis. Additionally, the calibration curve evaluated the nomogram’s performance of SMC4, and the Cindex of OS was 0.715 (**Figure 9H**). Moreover, recent research showed that the down-regulation of SMC4 expression could significantly inhibit the proliferation of colon cancer cells and reduce their malignancy. It is inferred that SMC4 may contribute to the occurrence of CRC and provide a new therapeutic target for the treatment of CRC (31). Furthermore, low expression levels of microRNA-124-5p can lead to a poor prognosis of CRC *via* targeting of SMC4, which further confirms the novel tumor prognostic marker and potential tumor therapeutic target role of SMC4 in CRC (32).

### 4.5 SMC4 and pancreatic cancer

**Figure 10:**
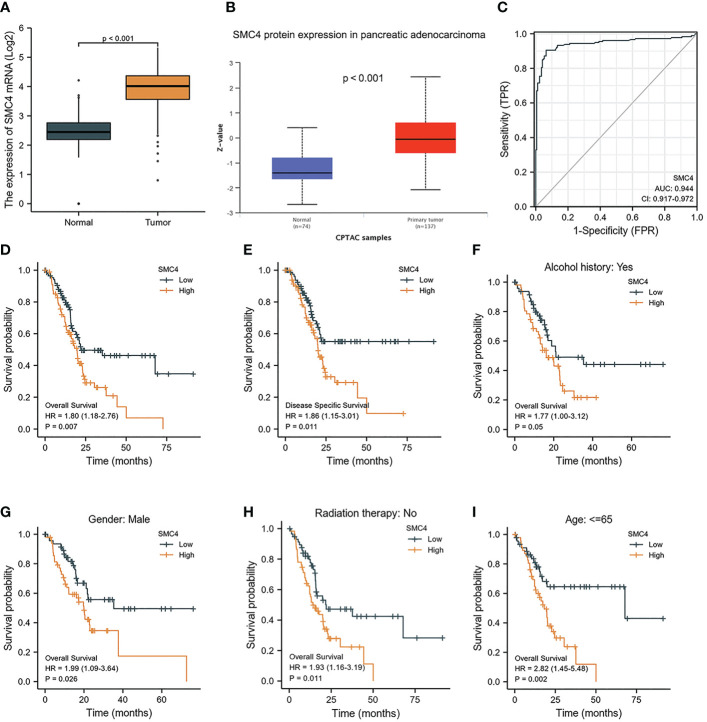
Expression levels of SMC4 and the prognostic values of SMC4 expression in PA patients. **(A)** Expression of SMC4 mRNA in PA and non-matched normal tissues in the TCGA databases. **(B)** Expression of SMC4 protein in PA in the CPTAC databases. **(C)** ROC curves for classifying PA versus normal pancreatic tissues in the TCGA database. **(D, E)** The prognostic value of SMC4 in OS and DSS of PA in the TCGA database. **(F–I)** OS survival curves of alcohol history, gender, radiation therapy, and age between high and low SMC4 patients with PA. TCGA, The Cancer Genome Atlas; OS, overall survival; DSS, disease-specific survival.

The main reasons for the poor prognoses of pancreatic adenocarcinoma (PA) patients are rapid early-stage progression, chemotherapy resistance, and advanced-stage metastasis. Identification of novel diagnostic and prognostic biomarkers of PA is therefore urgently needed. Recently, Zuo D et al. first observed that SMC4 was associated with immune cell infiltration in the tumor microenvironment (TME) of PA using Gene Ontology (GO) and Kyoto Encyclopedia of Genes and Genomes (KEGG) pathway enrichment analyses, where its mechanism may be related to that immune cell infiltration, and secretion of cytokine promote the formation of the TME in PA, thus facilitating tumor progression, metastasis, and therapeutic resistance (33). In addition, bioinformatics analysis manifested that the expression of SMC4 mRNA and protein was significantly higher in PA tissues than in normal tissues (*p* < 0.001) (**Figures 10A, B**). The ROC curve also suggested that SMC4 expression had good predictive power with an area under the curve (AUC) of 0.944 (95% CI: 0.917–0.972) to discriminate PA tissues from normal tissues (**Figure 10C**). PA patients were divided into high and low expression groups based on the SMC4 expression median value. The high expression group has a strong correlation with worse OS (HR = 1.80 (1.18–2.76), *p* = 0.007) and DSS (HR = 1.86 (1.15–3.01), *p* = 0.011) (**Figures 10D, E**). Furthermore, the prognosis of patients with high SMC4 expression was notably more unfavorable in several subgroups, including alcohol history (HR = 1.77 (1.00–3.12), *p* = 0.05), gender (HR = 1.99 (1.09–3.64), *p* = 0.026), radiation therapy (HR = 1.93 (1.16–3.19), *p* = 0.011) and age (HR = 2.82 (1.45–5.48), *p* = 0.002) (**Figures 10F–I**). Additionally, a study by Fukuhisa H et al. demonstrated that SMC4 gene was identified as a possible oncogenic target by miR-130b-5p regulation in PA cells and then affected the proliferation, migration, and invasion of PA (34). These results indicate that SMC4 can be reported as a novel diagnostic or even therapeutic marker for future studies in PA (35).

### 4.6 SMC4 and cervical cancer

Cervical cancer is one of the most common malignancies associated with mortality in females. Its onset and prognosis are primarily concerned with persistent infection with high-risk types of human papillomavirus (HPV). However, the definite molecular mechanisms of cervical cancer remain unclear. High-throughput sequencing to identify differentially expressed miRNAs and three series were selected from the Gene Expression Omnibus database to identify differentially expressed genes. Then the miRNA-TF-gene regulatory network was constructed using bioinformatic methods. Genes in the network were performed functional enrichment analysis and protein-protein interaction network analysis. The results show that SMC4 may be recognized as one hub gene with the highest connectivity degrees and be suggested to play crucial roles in the development of HPV-positive cervical cancer (36). In the Cox regression model, univariate Cox regression indicates that the T stage (*p* = 0.025), N stage (*p* = 0.002), M stage (*p* = 0.023), and SMC4 (*p* = 0.043) were correlated with the bad prognosis of cervical cancer. All variables in univariate Cox regression were included in multivariate Cox regression. Multivariate Cox regression showed that N stage (*p* = 0.047) was an independent prognostic factor for OS (**Table 3**). Moreover, further research by protein-protein interaction network analysis showed that SMC4 was closely associated with MELK and TTK, which were promising candidate markers for cervical cancer prognosis and also emerged as potential therapeutic drug targets (37). *In vitro*, cytological studies also demonstrated that SMC4 overexpression facilitated the development of cervical cancer cells by activating NF-κB pathway, which provides a new therapeutic target for patients with cervical cancer (38).

**Table 3:** Univariate and multivariate analyses of clinical pathological parameters in patients with cervical cancer from TCGA database.

Characteristics	Total (N)	Univariate analysis		Multivariate analysis	
		Hazard ratio (95% CI)	*P* value	Hazard ratio (95% CI)	*P* value
T stage	243				
T1	140	Reference			
T2&T3&T4	103	1.906 (1.085-3.348)	**0.025**	0.790 (0.223-2.805)	0.716
N stage	195				
N0	134	Reference			
N1	61	2.844 (1.446-5.593)	**0.002**	3.424 (1.017-11.520)	**0.047**
M stage	127				
M0	116	Reference			
M1	11	3.555 (1.187-10.641)	**0.023**	0.000 (0.000-Inf)	0.998
Clinical stage	299				
Stage I	162	Reference			
Stage II&Stage III&Stage IV	137	1.462 (0.920-2.324)	0.108		
SMC4	306	1.091 (0.879-1.354)	**0.043**		
Age	306				
<=50	188	Reference			
>50	118	1.289 (0.810-2.050)	0.284		
Birth control pill history	158				
No	89	Reference			
Yes	69	0.673 (0.324-1.397)	0.288		

Bold values means statistically significant (p < 0.05).

### 4.7 SMC4 and hematological tumors

Research on SMC4 in hematological tumors is scarce. At present, it can be reviewed only in areas of acute myeloid leukemia (AML) and acute lymphoblastic leukemia (ALL). Researchers established an AML mouse model induced by MLL-AF9 and found that down-regulating the expression of SMC4 can prolong the survival time of AML mice in the study. Knockout of SMC4 expression can reduce the proportion of leukemia stem cell (LSC) and then affect its ability to start leukemia. Cell cycle analysis also showed that SMC4 gene knockout could inhibit more LSCs in G0 phase of cell differentiation (39). It further revealed that SMC4 played a vital role in the progression of AML and provided new insights into the maintenance mechanism of leukemia stem cells. Clinical research also demonstrated that the expression of SMC4 was associated with various clinical outcomes in pediatric patients with ALL; the high SMC4 expression group had a worse disease-specific survival rate and poorer overall survival rate (40).

### 4.8 SMC4 and glioma

**Figure 11:**
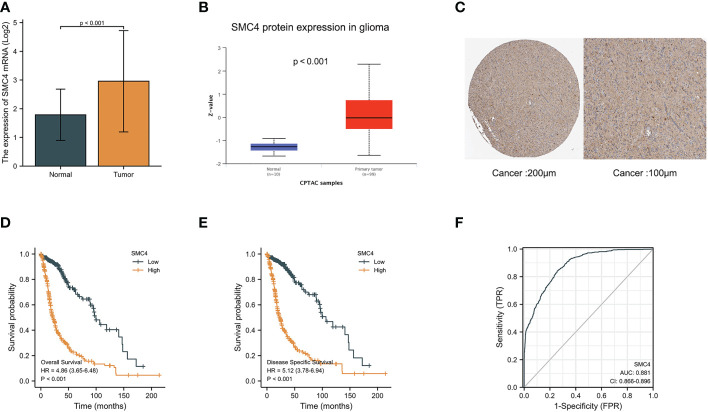
Expression levels of SMC4 mRNA and SMC4 protein in glioma and the prognostic value of SMC4 in glioma. **(A)** Expression of SMC4 mRNA level in glioma and non-matched normal tissues in the TCGA database. **(B)** Expression of SMC4 protein level in glioma in the CPTAC database. **(C)** SMC4 protein level in glioma using immunohistochemistry was analyzed using The Human Protein Atlas database. **(D)** Overall survival (OS) and **(E)** disease-specific survival (DSS) for glioma patients with high versus low SMC4. **(F)** ROC curves for classifying glioma versus normal tissues in the TCGA database. TCGA, The Cancer Genome Atlas; CPTAC, Clinical Proteomic Tumor Analysis Consortium; ROC, receiver operating characteristic.

Glioma is a common primary malignant brain tumor characterized by high mortality and poor prognosis. Bioinformatics analysis based on TCGA and CPTAC databases indicated that the expression of SMC4 mRNA and protein was significantly higher in glioma than in normal tissues (*p* < 0.001) (**Figures 11A, B**). The immunohistochemistry results from The Human Protein Atlas database also showed that SMC4 protein level was clearly enhanced in glioma (**Figure 11C**). The correlation between SMC4 expression and the prognosis of patients with glioma was calculated using the Kaplan Meier method. The median value of SMC4 expression was used as a cut-off score, and the patients were divided into high and low SMC4 expression groups. Compared with the low SMC4 expression group, both the OS (HR = 4.86 (3.65–6.48, *p* < 0.001) and DSS (HR = 5.12 (3.78–6.94, *p* < 0.001) of the high SMC4 expression group exhibited a significantly worse prognosis (**Figures 11D, E**). ROC showed that the expression of SMC4 mRNA in glioma was 0.881 (95% CI: 0.866–0.896) (**Figure 11F**). Overexpression of SMC4 can significantly improve the proliferation rate, migration rate, and invasion ability of glioma cells *in vivo* and *in vitro*, while the down-regulation of SMC4 is just the opposite. Depth studies have shown that SMC4 overexpression through the activation of TGF β/Smad signaling promotes the invasive phenotype of glioma cells. This study provides a new insight into the cofunction of SMC4 and the mechanism by which the TGF β/Smad pathway is hyperactivated in gliomas, indicating that SMC4 is a valuable prognostic factor and a potential therapeutic target in gliomas (41). Through integrated bioinformatic analysis, SMC4 was also identified as a potential prognostic biomarker for low-grade glioma patients, which functions to promote cell proliferation by repairing replication damage, and the expression of SMC4 could be transcriptionally regulated by MYB or directly by miR-433-3p (42, 43).

### 4.9 SMC4 and other tumors

Nasopharyngeal carcinoma (NPC) is a malignant tumor of the nasopharyngeal epithelium. Despite the therapeutic enhancements in the treatment of NPC, late diagnosis and metastasis influence survival and poor prognosis. Liu K et al. proposed that SMC4 may be involved in the tumorigenesis of NPC. Furthermore, it may be utilized as a molecular biomarker in the early diagnosis of NPC (44). Similar results also exist in the research field of prostate cancer, and bladder Carcinoma; SMC4 is demonstrated to be the potential biomarker and therapeutic target (45, 46).

## 5 Summary and prospect

Malignant tumors remain a significant cause of mortality worldwide and are the second leading cause of death in the United States (47). However, an invariable factor is that early diagnosis is crucial for the successful treatment of malignant tumors; therefore, it is necessary to explore and identify a new reliable tumor biomarker actively. As an ATP-sensitive switch, SMC4 protein, together with SMC2 monomer and other non-SMC subunits, forms a condensin. It plays a critical role in promoting the compression and dissociation of sister chromatids, DNA recombination, and repair after DNA damage. Along with RNA splicing, DNA metabolism, cell adhesion, and extracellular matrix, SMC4 is also extremely important for the division cycle of embryonic cells. Similar to how normal somatic cells can replicate the gene expression patterns of embryonic cells to gain an edge over them, tumor cells with high SMC4 expression may do the same (14, 48). Moreover, SMC4 is also a positive regulator of the inflammatory innate immune response, while An excessive innate immune response not only disrupts the immune balance, leading to autoimmune diseases but also even results in cancer. Combined with relevant kinds of literature and bioinformatics analysis, we found that SMC4 protein and its microRNA levels are abnormally expressed in a variety of tumor cells and tissues, and are involved in the occurrence and development of tumors, which is closely related to the proliferation, migration, and invasion of tumors. The high expression of SMC4 also predicts the poor prognosis of tumors. The correlation between SMC4 and tumors is realized through a variety of signal pathways (**Table 4**), which indicates that SMC4 can be used as a clinical treatment target for a variety of tumors, contribute to the individualized treatment of tumors, and improve the treatment results of tumors.

**Table 4:** Tumor effect of SMC4 and its possible mechanism or pathway.

Tumor Type	Tumor Effect (Y/N)	Mechanism or Pathway of Tumor Effect
primary liver cancer	Y	miR-219-SMC4-JAK2/STAT3 (17) HIF-1-miR-219-SMC4 (18)
lung cancer	Y	Cell cycle, cell adhesion and RNA processing (14)
breast cancer	Y	p53 network (24)
colorectal cancer	Y	LINC-ROR/miR-6833-3p/SMC4 network (30) microRNA-124-5p-SMC4 (32)
pancreatic cancer	Y	miR-130b-5p-SMC4 (34)
cervical cancer	Y	TTK (Positive regulation of cell proliferation), MELK (G2/M cell cycle) (37) NF-κB pathway (38)
hematological tumors	Y	inhibition of cell differentiation in G0 phase (39)
glioma	Y	TGF β/Smad pathway (41) MYB/miR-433-3p-SMC4 (42, 43)
Others	Y	has_circTPT1_003-has-miR-218-5p-CCNE2/SMC4 signaling axis (44)

Y/N, Yes/No.

In conclusion, we revealed that compared with the normal tissues, the expression of SMC4 was significantly up-regulated in many malignant tumor tissues. It plays an essential role in the occurrence and development of tumors, which is closely related to the proliferation, migration, and invasion of tumors. Understanding the function of SMC4 in tumor progression will not only advance our knowledge of the mechanisms underlying tumor aggressiveness, but also establish SMC4 as a novel tumor prognostic marker or a potential therapeutic target for treating tumors.

## Ethics approval

The study was approved by the Institutional Ethics Committee of Binzhou Medical University Hospital, Shandong Province, China. All procedures performed in studies involving human participants were in accordance with the ethical standards of the institutional and national research committee and with the 1964 Helsinki Declaration and its later amendments or comparable ethical standards.

## Author contributions

ZZ and XC are the guarantors of the manuscript and contributed to conception and design of the study, acquisition and analysis of data, and writing and revision of the manuscript. XW and YD collected data and analyzed statistics, XC and XZ provided guidance and funding. All authors contributed to the article and approved the submitted version.
